# Downregulation of Light-Harvesting Complex II Induces ROS-Mediated Defense Against Turnip Mosaic Virus Infection in *Nicotiana benthamiana*

**DOI:** 10.3389/fmicb.2021.690988

**Published:** 2021-07-05

**Authors:** Shiyou Qiu, Xuwei Chen, Yushan Zhai, Weijun Cui, Xuhong Ai, Shaofei Rao, Jianping Chen, Fei Yan

**Affiliations:** ^1^College of Life Sciences, Fujian Agriculture and Forestry University, Fuzhou, China; ^2^State Key Laboratory for Managing Biotic and Chemical Threats to the Quality and Safety of Agro-products, Institute of Plant Virology, Ningbo University, Ningbo, China; ^3^Key Laboratory of Biotechnology in Plant Protection of MOA of China and Zhejiang Province, Institute of Virology and Biotechnology, Zhejiang Academy of Agricultural Sciences, Hangzhou, China

**Keywords:** turnip mosaic virus, TuMV, *LHCB3*, reactive oxygen species, ROS

## Abstract

The light-harvesting chlorophyll *a*/*b* complex protein 3 (LHCB3) of photosystem II plays important roles distributing the excitation energy and modulating the rate of state transition and stomatal response to abscisic acid. However, the functions of LHCB3 in plant immunity have not been well investigated. Here, we show that the expression of *LHCB3* in *Nicotiana benthamiana* (*NbLHCB3*) was down-regulated by turnip mosaic virus (TuMV) infection. When *NbLHCB3* was silenced by tobacco rattle virus-induced gene silencing, systemic infection of TuMV was inhibited. H_2_O_2_ was over-accumulated in *NbLHCB3*-silenced plants. Chemical treatment to inhibit or eliminate reactive oxygen species (ROS) impaired the resistance of the *NbLHCB3*-silenced plants to TuMV infection. Co-silencing of *NbLHCB3* with genes involved in ROS production compromised the resistance of plants to TuMV but co-silencing of *NbLHCB3* with genes in the ROS scavenging pathway increased resistance to the virus. Transgenic plants overexpressing *NbLHCB3* were more susceptible to TuMV. These results indicate that downregulation of *NbLHCB3* is involved in defense against TuMV by inducing ROS production.

## Introduction

Chloroplasts are not only the organelles where photosynthesis takes place, but also the biosynthetic site of defense-related molecules like reactive oxygen species (ROS) and phytohormones like jasmonic acid (JA) (Fonseca et al., 2009) and salicylic acid (SA) (Strawn et al., 2007). ROS are able to kill pathogens directly by oxidation. The accumulation of ROS is known as the pathogen-associated molecular pattern (PAMP)-triggered immunity (PTI) response (Park et al., 2012). Increasing evidence shows that ROS produced by plant plasma membrane respiratory burst oxidase homologs (RBOHs) act as secondary messages which are involved in different signal transduction pathways triggering a physiological or programmed pathway for cell death (Baxter et al., 2014; Schieber and Chandel, 2014; Adachi et al., 2015; Waszczak et al., 2018). Recently Wu et al. (2020) found that a leucine-rich-repeat receptor kinase named hydrogen-peroxide-induced Ca^2+^ increases (HPCA) is a sensor to perceive extracellular H_2_O_2_ and induce activation of Ca^2+^ channels to trigger an influx of Ca^2+^ ions in *Arabidopsis*.

To efficiently harvest solar energy, light-harvesting antenna protein complexes bind chlorophylls and carotenoids and then create a number of light-harvesting chlorophyll *a*/*b* binding proteins named LHCBs or LHCAs (Jansson, 1999; Klimmek et al., 2005, 2006). LHCB proteins are associated with the light-harvesting complexes of photosystem II (PS II), while LHCA proteins are associated with those of photosystem I (PS I). PS I is central to the light-driven conversion of water to molecular oxygen, and consists of a PS II core dimer (C_2_) bound by four trimeric LHCBs (LHCB1–3) that are surrounded by the monomeric LHCB4 (CP29), LHCB5 (CP26), and LHCB6 (CP24) (Caffarri et al., 2009). By modulating the rate of state transitions, LHCB3 is involved in excitation energy transfer and charge separation and is also required for stomatal response to abscisic acid (Damkjaer et al., 2009; Xu et al., 2012; Adamiec et al., 2015). In Arabidopsis, LHCB3 affects the macrostructure of photosystem II and the rate of state transitions (Damkjaer et al., 2009; Kouřil et al., 2013; Pietrzykowska et al., 2014; Longoni et al., 2015), and in tomato and oil palm, the *LHCB3* gene confers continuous light tolerance and enhances yield (Velez-Ramirez et al., 2014; Neoh et al., 2019). LHCB3 is involved in cyclic electron flow and then affects state transition by interacting with PGR5a in cucumber (Wu et al., 2021b). However, it is not known whether LHCB3 functions in plant immunity.

We now report that the expression of *Nicotiana benthamiana LHCB3* (*NbLHCB3*) is downregulated in plants infected with turnip mosaic virus (TuMV). When *NbLHCB3* was silenced, systemic infection of TuMV was inhibited. Further analysis showed that the accumulated ROS in the *NbLHCB3*-silenced plants form an essential part of the plant defense against TuMV infection. The results indicate a mechanism in which the downregulation of *NbLHCB3* is involved in defense against TuMV by inducing ROS.

## Materials and Methods

### Plant Materials and Growth Conditions

*Nicotiana benthamiana* plants were grown in pots with premixed soil in a climate chamber at 26°C, with 60% humidity and under a 16-/8-h photoperiod (light daily from 08:00 to 24:00) with an average light intensity of 130 μmol m^–2^ s^–1^.

### Viral Inoculation and Detection

Agroinfiltration was done as described before (Li et al., 2019) using *Agrobacterium tumefaciens* transformed with viral infectious clones infiltrated into *N. benthamiana* leaves at the stage when three or five true leaves were fully expanded. For mechanical inoculation, *N. benthamiana* leaves previously infiltrated with agrobacteria carrying an infectious clone and displaying viral symptoms were homogenized in 0.1 M phosphate buffer (pH 7.2) using a sterile pestle and mortar and then filtered. Standardized volumes of infectious sap (or phosphate buffer for controls) were then applied to the upper surface of fully expanded leaves which had been sprayed with carborundum. The distribution of virus in infected tissues or plants was first determined by the intensity of visible green fluorescence under a handheld UV lamp. The transcriptional or translational levels of viral capsid protein were then determined by qRT-PCR, northern blot and western blot using the procedures described below.

### Agrobacterium-Mediated Transient Expression in *N. benthamiana* Leaves

The cDNA fragment of *NbLHCB3* was cloned into expression vectors with different tags using ligation-independent cloning (LIC) exactly as described previously (Wang et al., 2019). Recombinant clones were transformed into Agrobacterium GV3101 by electroporation, grown at 28°C overnight, collected by centrifugation, resuspended in infiltration buffer [10 mM MgCl_2_, 10 mM 2−(*N*−morpholino) ethanesulfonic acid (MES), and 200 μM acetosyringone, pH 5.6] and kept at room temperature for 4 h. Fully expanded true leaves of *N. benthamiana* were infiltrated with *A. tumefaciens* resuspension solution (OD_600_ = 1.0) and were harvested at 36∼72 hours post-infiltration (hpi) for further research. For co-expression, equal concentrations and equal volumes of individual Agrobacterium cultures were mixed.

### Virus-Induced Gene Silencing

The tobacco rattle virus (TRV)-based virus-induced gene silencing (VIGS) system was used to silence genes in *N. benthamiana*. This system includes TRV vectors, pYL196 (TRV1) and pYL156 (TRV2), both of which were kindly provided by Prof. Yule Liu at Tsinghua University (Beijing, China) (Liu et al., 2002). Specific primers were designed and used to amplify a ∼300 bp cDNA fragment of the target gene from the *N. benthamiana* cDNA library. For virus-induced double gene silencing, two fragments were amplified and then linked by homologous recombination. The fragment was then cloned into the vector pYL156 by the LIC method (Wang et al., 2019) to yield the VIGS vector. The negative control vector was prepared using a fragment of the gene encoding glucuronidase. Equal volumes of *Agrobacteria* carrying pYL196 and pYL156 constructs at OD_600_ = 1 were mixed and co-infiltrated into *N. benthamiana* plants. At least 12 plants were used for each silencing treatment and the same number of plants were used as controls. The infiltrated plants were returned to the climate chamber and used for further analysis at about 15 days post-infiltration (dpi).

### Total RNA Extraction and RNA Analysis

Total RNA was extracted from leaf tissues of *N. benthamiana* using Trizol reagent (Thermo Fisher, Waltham, MA, United States) as instructed. DNA contamination was removed and cDNA synthesized using ReverTra Ace^®^ qPCR RT Master Mix with gDNA Remover (TOYOBO, Japan). Real-time PCR was performed in a 10 μL volume containing 2 μL of 25-fold diluted cDNA, 5 μM each primer, and 1 × SYBR Green PCR Mix (Vazyme, China) in a LightCycler®480 II Real-Time PCR System (Roche, Switzerland) and the results were analyzed by the ΔΔCT method (Livak and Schmittgen, 2001). The *N. benthamiana* Ubiquitin C gene (*UBC*, AB026056.1) (Rotenberg et al., 2006; Shi et al., 2016) was used as an internal reference for analysis. Four individual plants per treatment were sampled and each treatment was repeated three times independently.

Northern blot of TuMV RNAs was performed as described before (Wang et al., 2019). The genomic RNA of TuMV was detected by a 260-bp fragment of TuMV CP gene labeled with digoxin (DIG) according to the DIG Oligonucleotide 3′-End Labeling Kit protocol (Roche, Switzerland). Ten micrograms of total RNA per sample was loaded on 1.5% formaldehyde-agarose gels and was separated by electrophoresis at 50 V for about 2 h in 1 × MOPS running buffer, and blotted to Hybond-NX nylon membranes (Amersham Biosciences, United Kingdom) for more than 16 h by capillary transfer. Subsequent procedures including pre-hybridization and hybridization were done following the protocol of the DIG High Primer DNA Labeling and Detection Starter Kit II (Roche).

### Western Blot

Equal weight samples of *N. benthamiana* leaves were ground to powder in liquid nitrogen, then resuspended in lysis buffer [100 mM Tris–HCl (pH 8.8), 6% sodium dodecyl sulfate (SDS), 2% β-mercaptoethanol] and centrifuged at 4°C for 12,000 rpm. Total proteins in the supernatants were separated in 12% SDS-PAGE (TGX Stain-Free FastCast Acrylamide Kit, Bio-Rad) and transferred onto 0.45 μm nitrocellulose membrane (Amersham Biosciences, United Kingdom) using semi-dry electroblotting, then incubated with relevant primary antibodies including anti-NbLHCB3 (YouLong Biotech, China), anti-TuMV CP (NEOGEN, United Kingdom), anti-GFP (Transgen Biotech, China) and anti-c-Myc (Gene Tex, Irvine, CA, United States). Secondary antibodies were AP-linked anti-rabbit or anti-mouse (Sigma-Aldrich, St Louis, MO, United States). The antigen-antibody complex was visualized by adding nitrotetrazolium blue chloride/5-bromo-4-chloro-3-indolyl phosphate (NBT/BCIP) (Sigma-Aldrich) at room temperature for a few minutes and results were captured with a Canon digital camera.

### ROS Detection and Treatment With Chemical Inhibitors

To detect H_2_O_2_ or O_2_^–^ by histochemical staining, six leaves of each plant were collected at 22:00, immersed and vacuum-infiltrated in freshly prepared solutions of either 3,3′-diaminobenzidine (DAB) [1 mg/ml DAB in Tris–HCl buffer (pH 3.8)] or NBT [0.2% (W/V) NBT in 50 mM PBS (pH 7.5)]. After incubation overnight in darkness at room temperature they were destained in 95% (W/V) ethanol by heating in a boiling water-bath to remove the chlorophyll. Images were captured with a Canon digital camera.

Diphenylene iodonium (DPI) (Mei et al., 2017; Zhang et al., 2020) is a chemical inhibitor of RBOHs. A concentrated stock solution of DPI (10 mM) was prepared by adding 10 mg DPI to 3.179 ml anhydrous dimethylsulphoxide (DMSO). This stock solution was then diluted with Agrobacterium infiltration buffer to provide a final working concentration of 50 μM for infiltration into *N. benthamiana* leaves.

Dimethylthiourea (DMTU) (Jiang and Zhang, 2002; Li et al., 2016) is a chemical inhibitor of H_2_O_2_ and responsible for scavenging H_2_O_2_. A concentrated stock solution of DMTU (150 mM) was prepared by adding 0.1563 g DMTU to 10 ml ddH_2_O. This stock solution was then diluted with Agrobacterium infiltration buffer to provide a final working concentration of 5 mM for infiltration into *N. benthamiana* leaves.

## Results

### The Expression of *NbLHCB3* Was Downregulated in TuMV-Infected *N. benthamiana*

**FIGURE 1:**
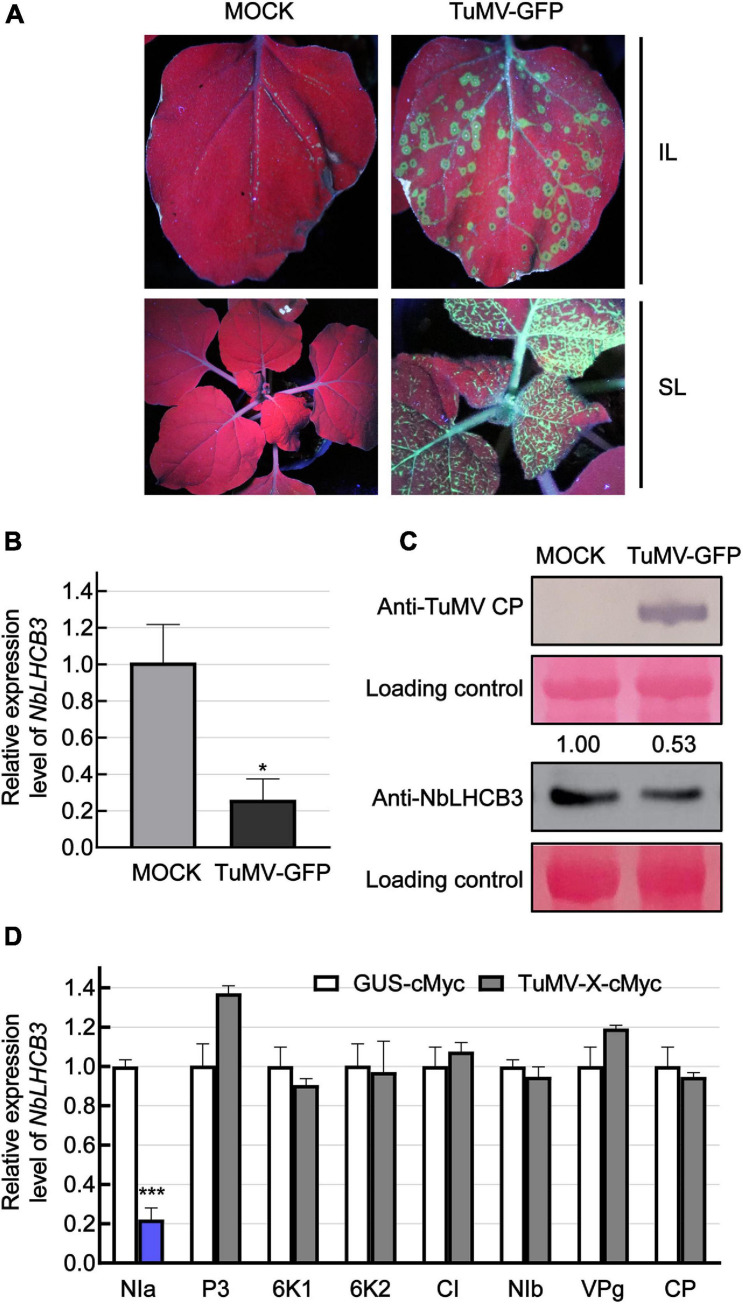
Expression of *NbLHCB3* was downregulated in *N. benthamiana* infected with TuMV-GFP. **(A)** Fluorescence under UV showing TuMV-GFP infection on inoculated leaves (IL) and systemically infected leaves (SL). Leaves of 2-week-old plants were mechanically inoculated with an extract of TuMV-GFP-infected leaves. Phosphate buffer was used as mock control. Pictures were captured at 4 dpi under UV. **(B)** qRT-PCR showing the transcript levels of *NbLHCB3* in the inoculated leaves of three mock and three TuMV-GFP-infected *N. benthamiana* plants at 4 dpi. Experiments were repeated three times. **(C)** Western blot analysis showing TuMV CP and NbLHCB3 accumulation on the systemically infected leaves of TuMV-GFP-infected plants at 4 dpi. The relative intensity of the blot signal quantified by IMAGE J software is shown on the lanes. **(D)** Results of qRT-PCR showing *NbLHCB3* expression in zones from four leaves of four plants expressing different cMyc-fused TuMV proteins. cMyc-fused beta-glucuronidase protein (GUS-cMyc) was used as control. Bars represent the standard errors of the means from three biological repeats. A two-sample unequal variance directional Student’s *t*-test was used to test the significance of the differences (*P < 0.05; ***P < 0.001).

In our previous work, we described the transcriptome of *N. benthamiana* plants infected with TuMV (Wu et al., 2021a). One gene with downregulated expression had the highest identity to *AtLHCB3*. For further analysis, we therefore first identified this gene from the genomic sequence of *N. benthamiana*. Using *AtLHCB3* (At5G54270) as query, *NbLHCB3* (Sequence ID Niben101Scf07510g00016.1) was identified in the *Solanaceae* Genomics Network.^1^ It was predicted to encode a protein with 265 amino acids. *NbLHCB3* had 88.8% protein identity and 79.9% nucleotide identity to *AtLHCB3* (Supplementary Figure 1). To confirm its expression response to TuMV infection, *N. benthamiana* leaves were mechanically inoculated with GFP-labeled TuMV so that viral infection could easily be monitored by detecting GFP fluorescence (Figure 1A). At 4 dpi, the inoculated leaves (IL) were collected and the expression of *NbLHCB3* was determined (Figure 1). There was a significant decrease in accumulation of both *NbLHCB3* transcripts and NbLHCB3 protein (Figures 1B,C) compared to mock-inoculated controls, suggesting that *NbLHCB3* was downregulated in the TuMV-infected plants.

To determine which viral protein is responsible for downregulated expression of *NbLHCB3*, we transiently expressed cMyc-fused TuMV proteins individually by agroinfiltration and detected the expression level of *NbLHCB3* in leaves at 2 dpi. In infiltrated leaf areas or regions expressing NIa-cMyc, expression of *NbLHCB3* was downregulated significantly, but not in those areas or regions expressing the other TuMV proteins, indicating that TuMV NIa is responsible for downregulation of *NbLHCB3* in the infected plants (Figure 1D).

### Silencing of *NbLHCB3* Reduced the Infection of TuMV in *N. benthamiana*

**FIGURE 2:**
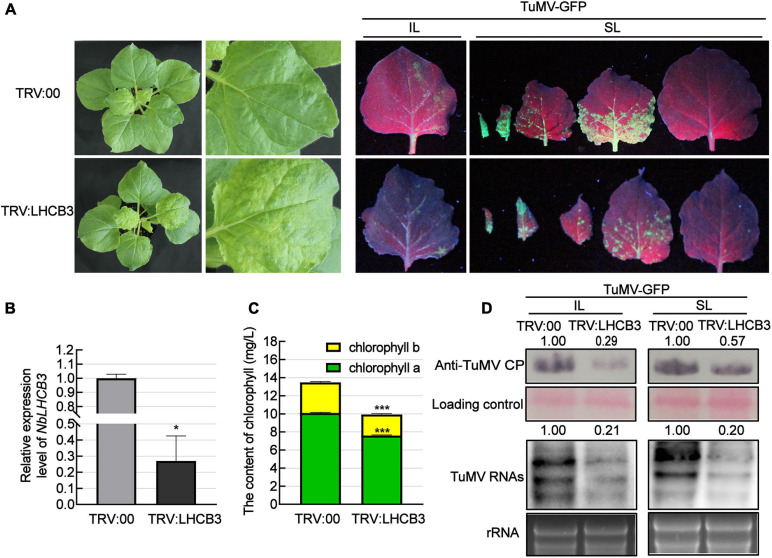
Silencing of *NbLHCB3* inhibited the infection of TuMV in *N. benthamiana*. **(A)** The phenotype of plants inoculated with TRV:LHCB3 or TRV:00 (Left). Examples of LHCB3-silenced and control plants mechanically inoculated with TuMV-GFP at 12 dpi and photographed at 4 dpi under UV light (Right). Systemically infected leaves (SL) at the corresponding location on both plants were collected for monitoring under UV. **(B)** Relative expression level of *NbLHCB3* in the *NbLHCB3*-silenced plants. **(C)** The chlorophyll contents [measured as described before (Yang et al., 2020)] were reduced in the *NbLHCB3*-silenced plants. **(D)** Detection of TuMV RNAs and CP protein accumulation in plants inoculated with TRV:LHCB3 or TRV:00 at 4 dpi. Polyclonal antibody to TuMV CP and a CP probe tagged with DIG were used for analysis. Bars represent the standard errors of the means from three biological repeats. The relative intensity of the blot signal quantified by IMAGE J software is shown on the lanes. A two-sample unequal variance directional Student’s *t*-test was used to test the significance of the differences (*P < 0.05; ***P < 0.001).

To investigate the function of *NbLHCB3* in plants infected by TuMV, we used the TRV-induced gene silencing system (VIGS), creating a VIGS vector (TRV:LHCB3) to silence *NbLHCB3* in at least 12 plants and then mechanically inoculated these plants with TuMV-GFP. At 12 dpi of TRV:LHCB3, the expression of *NbLHCB3* was decreased to 30% of the normal (TRV: 00 control) level (Figures 2A,B). We also determined the expression of other members of the *NbLHCB* family in the TRV:LHCB3-infected plants but found no obvious difference in their expression compared to that in the control TRV:00-infected plants, indicating that *NbLHCB3* had been specifically silenced in the TRV:LHCB3-infected plants (Supplementary Figure 2). The silenced plants showed mild yellowing with reduced chlorophyll content (Figures 2A,C). The plants inoculated with TRV:LHCB3 and TRV:00 were then mechanically inoculated with TuMV-GFP. At 4 dpi of TuMV-GFP, there were fewer fluorescent infection foci on the IL of the *NbLHCB3*-silenced plants than on non-silenced plants (Figure 2A). TuMV RNAs and CP protein in both IL and systemically infected leaves accumulated less in the *NbLHCB3*-silenced plants than in non-silenced plants (Figure 2D), showing that silencing of *NbLHCB3* inhibited TuMV-GFP infection.

### ROS Accumulated in the *NbLHCB3*-Silenced Plants

**FIGURE 3:**
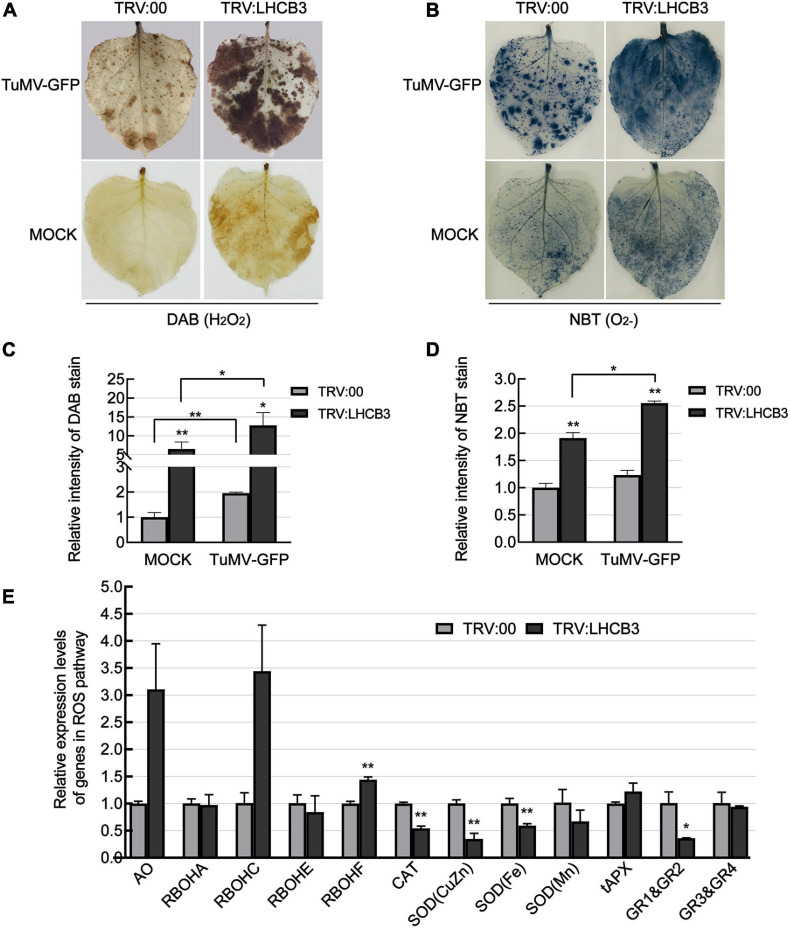
Reactive oxygen species accumulated in the *NbLHCB3*-silenced plants. **(A)** Detection of H_2_O_2_ by DAB staining in plants inoculated with TRV:LHCB3 or TRV:00 with or without TuMV infection. **(B)** Detection of O_2_^−^ by NBT staining in the plants inoculated with TRV:LHCB3 or TRV:00 with our without TuMV infection. **(C)** The relative DAB stain intensity of the *NbLHCB3*-silenced and control plants. **(D)** The relative NBT stain intensity of the *NbLHCB3*-silenced and control plants. **(E)** Identification of transcriptional levels of genes in ROS production and scavenging pathways. The relative DAB and NBT stain intensity was quantified by IMAGE J software. Bars represent the standard errors of the means from three biological repeats. A two-sample unequal variance directional Student’s *t*-test was used to test the significance of the differences (*P < 0.05; **P < 0.01).

Light-harvesting chlorophyll *a*/*b* binding proteins play a key role in the electron transport chain in chloroplasts, which are the major sites of ROS production (Galvez-Valdivieso and Mullineaux, 2010). In previous reports, ROS levels increased in all the *Arabidopsis thaliana lhcb* T-DNA insertion mutants (Xu et al., 2012). We next used DAB and NBT staining to determine the H_2_O_2_ and O_2_^–^ levels in the *NbLHCB3*-silenced plants before and after inoculation with TuMV. H_2_O_2_ and O_2_^–^ accumulated significantly in the *NbLHCB3*-silenced plants regardless of TuMV infection (Figures 3A–D). We also determined the expression of genes involved in ROS production or scavenging by qRT-PCR in the *NbLHCB3*-silenced plants and found that genes encoding AO (aldehyde oxidase) (Yergaliyev et al., 2016; Wu et al., 2017) and respiratory burst oxidase homolog C (RBOHC) (Pei et al., 2003) were all expressed more in the *NbLHCB3*-silenced plants (Figure 3E). By contrast, expression of genes encoding CAT (catalase) (Lee et al., 2017), SOD(CuZn) [superoxide dismutase (CuZn)], SOD(Fe), SOD(Mn) (Miller, 2012), and Glutathione reductase (GR) (Mittler et al., 2004), the major components of ROS scavenging systems, were all significantly downregulated (Figure 3E). These results demonstrate that ROS accumulated in the *NbLHCB3*-silenced plants.

### Suppressing ROS Accumulation Facilitated TuMV Infection in *NbLHCB3*-Silenced Plants

**FIGURE 4:**
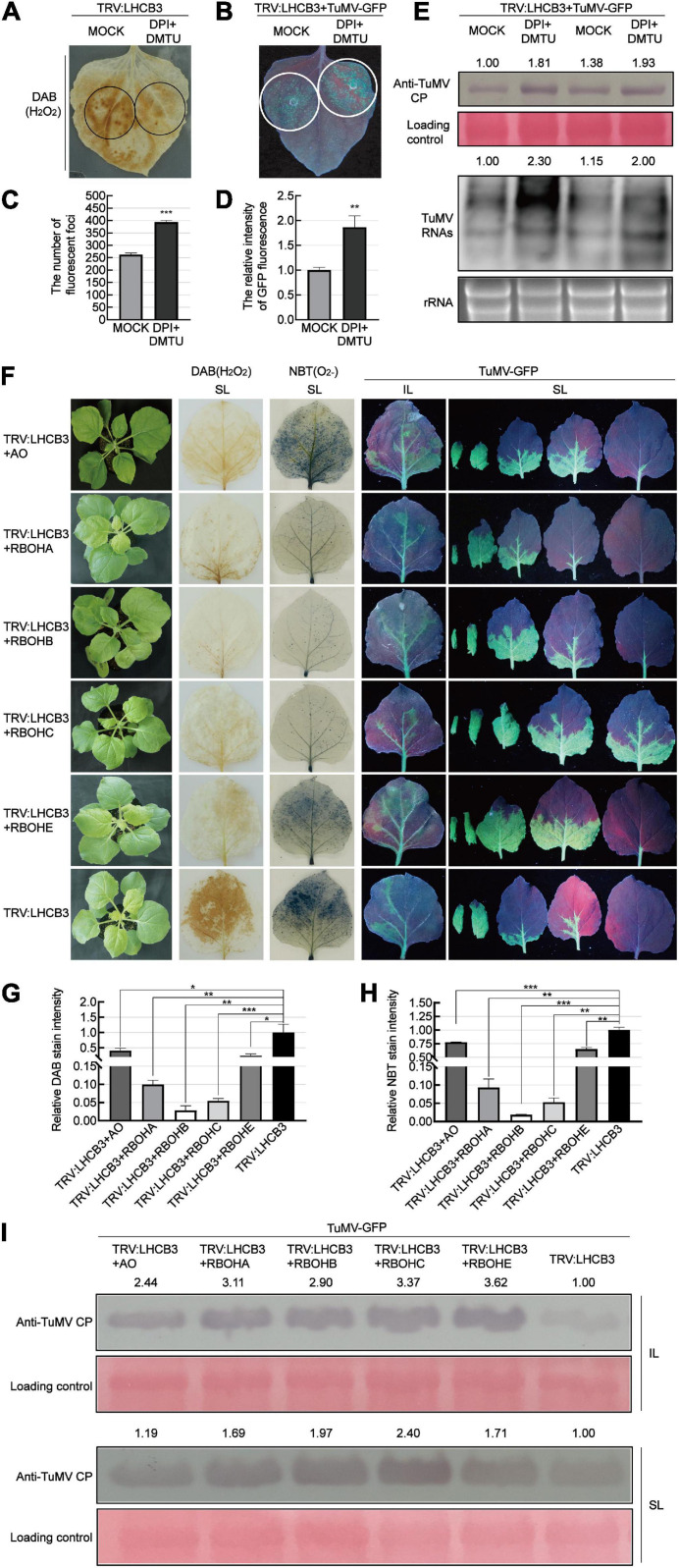
Suppressing ROS accumulation facilitated TuMV infection in the *NbLHCB3*-silenced plants. **(A)** DAB staining of ROS accumulation in the TRV:LHCB3 plants after being treated with DPI and DMTU. Treated zones are circled in black. **(B)** Treatment of DPI and DMTU facilitated TuMV-GFP infection in the *NbLHCB3*-silenced plants. Treated zones are circled in white. GFP fluorescence is shown under UV light at 4 dpi. **(C)** The number of fluorescent foci in the zones of *NbLHCB3*-silenced leaves treated or not with chemical inhibitors of ROS. **(D)** The relative intensity of GFP fluorescence in the zones of *NbLHCB3*-silenced leaves treated or not with chemical inhibitors of ROS. **(E)** Accumulation of TuMV CP protein and RNAs in the TRV:LHCB3 plants treated with DPI or DMTU. **(F)** TuMV-GFP infection in plants where *NbLHCB3* was co-silencing with different genes in the ROS production pathway. The phenotype of plants under bright light is shown at the left. DAB staining and NBT staining show ROS accumulation in the silenced plants. TuMV-GFP infection in the inoculated leaves (IL) and systemically infected leaves (SL) was monitored under UV light at 5 dpi. SL at the corresponding location in plants were collected for analysis. **(G)** The relative DAB stain intensity calculated by IMAGE J software. **(H)** The relative NBT stain intensity calculated by IMAGE J software. **(I)** Western blotting showing the accumulation of TuMV-GFP CP in the plants at 5 dpi. The relative intensity of the blot signal was quantified by IMAGE J software. Bars represent the standard errors of the means from three biological repeats. A two-sample unequal variance directional Student’s *t*-test was used to test the significance of the differences (*P < 0.05; **P < 0.01; ***P < 0.001).

To determine whether the accumulation of ROS plays a role against TuMV infection in the *NbLHCB3*-silenced plants, we treated the silenced plants with 50 μM DPI and 5 mM dimethylthiourea (DMTU) to suppress the production of ROS and eliminate existing ROS, and then examined its effect on TuMV infection (Figure 4). The amount of ROS in the *NbLHCB3*-silenced leaves was indeed decreased after being treated with DPI and DMTU (Figure 4A). When TuMV-GFP was inoculated, more fluorescent infection foci appeared and there was stronger GFP fluorescence on the IL of the *NbLHCB3*-silenced plants treated with DPI and DMTU, compared to those of the non-treated *NbLHCB3*-silenced plants (Figures 4B–D). Furthermore, TuMV RNAs and CP protein accumulated at higher levels in the *NbLHCB3*-silenced plants treated with DPI and DMTU than in the non-treated *NbLHCB3*-silenced plants (Figure 4E), indicating that the accumulated ROS in the *NbLHCB3*-silenced plants plays a role against TuMV infection.

To further confirm this, we co-silenced *NbLHCB3* and each of the individual genes involved in ROS production: *AO*, *RBOHA*, *RBOHB*, *RBOHC*, and *RBOHE*. The downregulated expression of these genes in the co-silenced plants was confirmed by qRT-PCR (Supplementary Figures 5A–E). It was expected that silencing of these genes would impair the accumulation of ROS in the *NbLHCB3*-silenced plants. Indeed, DAB and NBT staining showed a significantly lower accumulation of ROS in these co-silenced plants than in those where only *NbLHCB3* was silenced (Figures 4F–H). TuMV-GFP was then mechanically inoculated onto these plants. At 5 dpi of TuMV-GFP infection, GFP fluorescence was stronger on the systemic leaves (SL) of TRV:LHCB3 + AO, TRV:LHCB3 + RBOHA, TRV:LHCB3 + RBOHB, TRV:LHCB3 + RBOHC, and TRV:LHCB3 + RBOHE compared with the TRV:LHCB3 plants (Figure 4F). Consistently, TuMV RNAs and CP protein accumulated at higher levels in both the IL and SL of these co-silenced plants (Figure 4I and Supplementary Figure 3). These results further demonstrate that the accumulated ROS in the *NbLHCB3*-silenced plants plays a role against TuMV infection.

### Co-silencing of Genes in the ROS Scavenging Pathway With *NbLHCB3* Increased the Resistance of *NbLHCB3*-Silenced Plants to TuMV Infection

**FIGURE 5:**
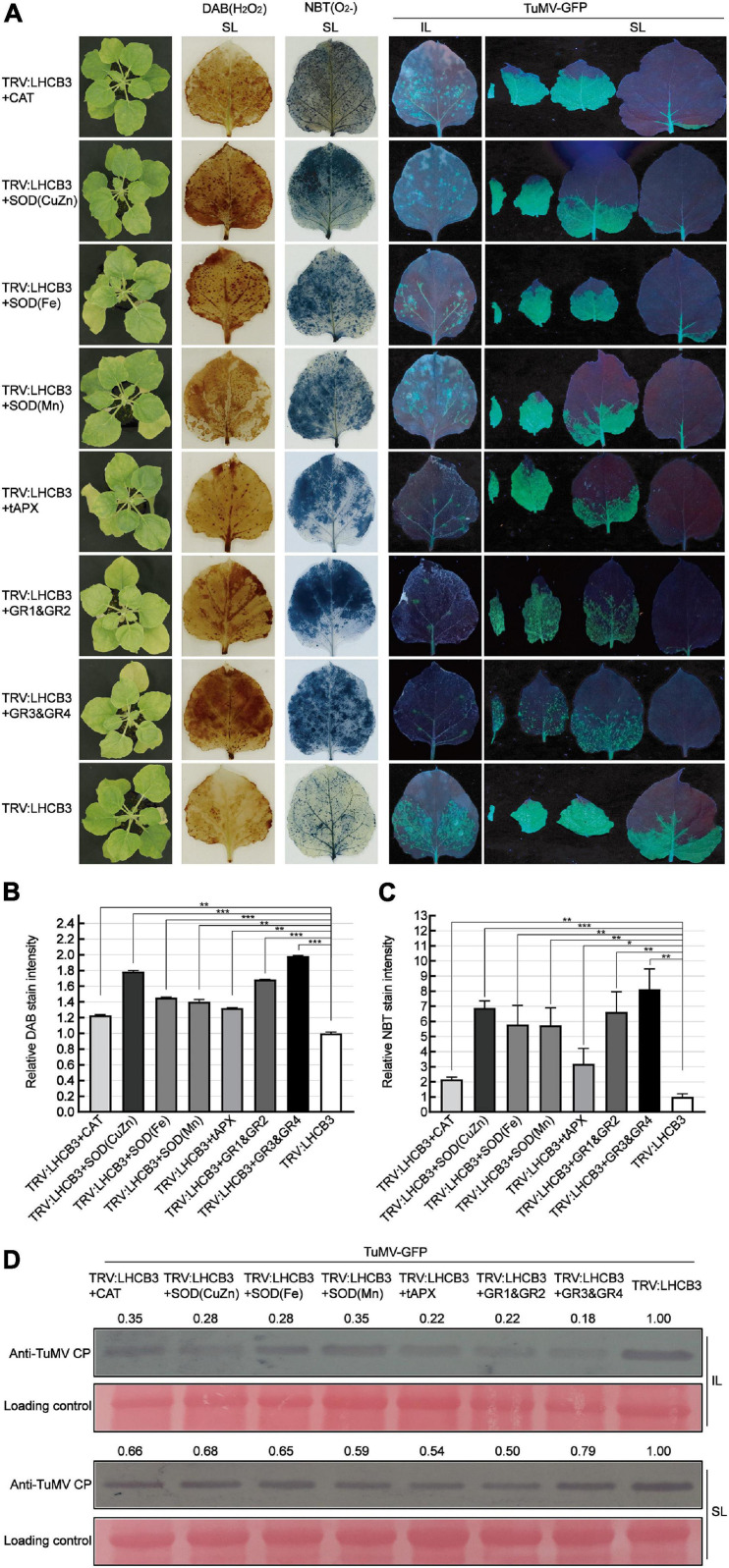
Co-silencing of *NbLHCB3* and genes in the ROS scavenging pathway further suppressed TuMV-GFP infection. **(A)** The phenotype of plants under bright light is shown at the left. DAB and NBT staining show ROS accumulation in the silenced plants. TuMV-GFP infection in the inoculated leaves (IL) and systemically infected leaves (SL) was monitored under UV light at 8 dpi. SL at the corresponding location in plants were collected for analysis. **(B)** The relative DAB stain intensity calculated by IMAGE J software. **(C)** The relative NBT stain intensity calculated by IMAGE J software. **(D)** Western blotting showed the accumulation of TuMV-GFP CP in the plants at 8 dpi. The relative intensity of the blot signal quantified by IMAGE J software is shown on the lanes. Bars represent the standard errors of the means from three biological repeats. A two-sample unequal variance directional Student’s *t*-test was used to test the significance of the differences (*P < 0.05; **P < 0.01; ***P < 0.001).

To further confirm the relationship between ROS and defense against TuMV in the *NbLHCB3*-silenced plants, we co-silenced genes in the ROS degradative pathway, *CAT* (CAT functions in H_2_O_2_ scavenging), *SOD*(*CuZn*), *SOD*(*Fe*), *SOD*(*Mn*) (SODs play pivotal roles in metabolizing O_2_^–^ and producing H_2_O_2_), *tAPX* (encodes ascorbate peroxidase which has a similar function to CAT and exists in thylakoids), *GR1*&*GR2* and *GR3*&*GR4*, with *NbLHCB3*, and examined TuMV infection on the silenced plants. The expression of these genes was shown to be significantly downregulated in the co-silenced plants by qRT-PCR (Supplementary Figures 5F–L). We expected that ROS would accumulate more in these co-silenced plants than in those where only *NbLHCB3* was silenced, and this was indeed confirmed by DAB and NBT staining (Figures 5A–C). TuMV-GFP was then inoculated onto these plants. At 8 dpi of inoculation, there were fewer fluorescent spots on the IL of TRV:LHCB3 + CAT, TRV:LHCB3 + SOD(CuZn), TRV:LHCB3 + SOD(Fe), TRV:LHCB3 + SOD(Mn), TRV:LHCB3 + tAPX, TRV:LHCB3 + GR1&GR2, and TRV:LHCB3 + GR3&GR4 plants than on the control TRV:LHCB3 plants. Consistently, fluorescent areas on the systemically infected leaves of these plants were small, while the fluorescence intensity was not affected significantly. TuMV RNAs and CP also accumulated at lower levels in these co-silenced plants (Figure 5D and Supplementary Figure 4). These results give further support to the conclusion that the accumulated ROS in the *NbLHCB3*-silenced plants plays roles against TuMV infection.

### Overexpression of *NbLHCB3* in *N. benthamiana* Increased the Accumulation of TuMV RNAs and CP

**FIGURE 6:**
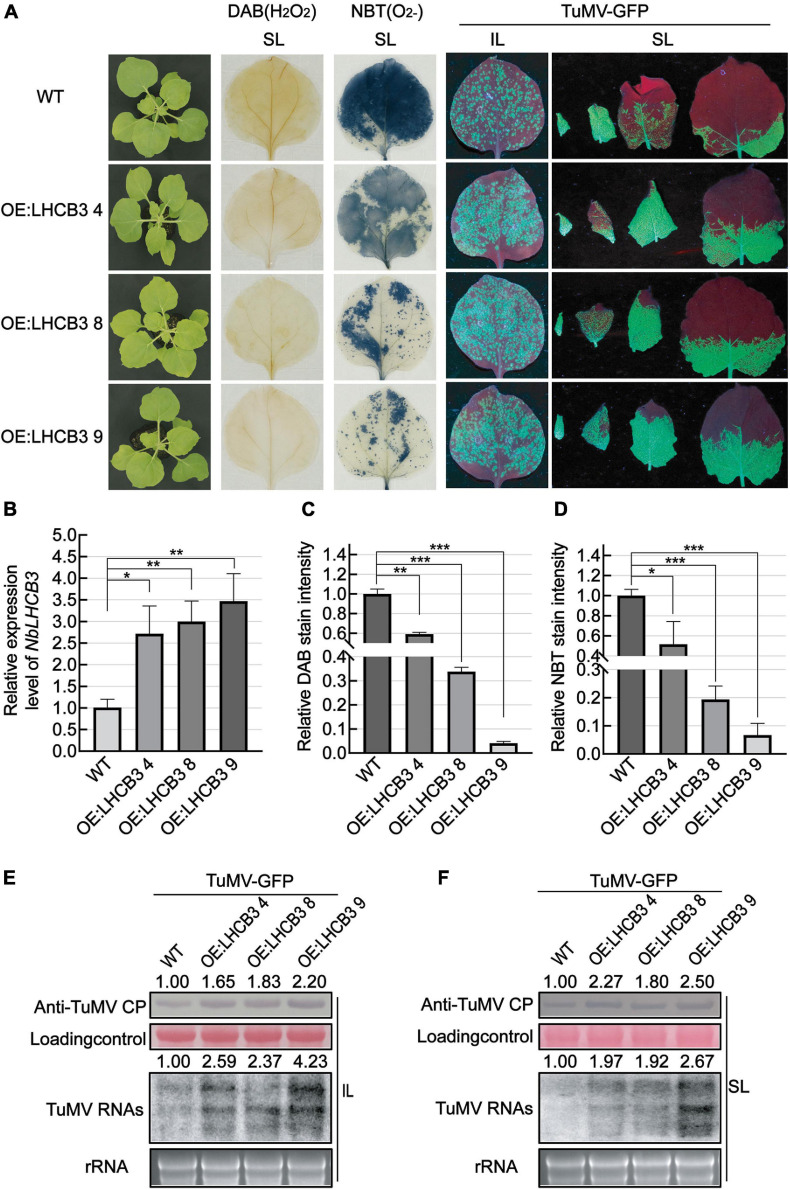
Overexpression of *NbLHCB3* in *N. benthamiana* increased the accumulation of TuMV RNAs and CP. **(A)** The phenotype of plants under bright light is shown at the left. DAB and NBT staining show less ROS accumulation in the transgenic plants. Fluorescence arising from TuMV-GFP infection on three transgenic lines and wild type (WT) plants: IL and SL were photographed at 5 dpi under UV light. **(B)** qRT-PCR assay of expression of *NbLHCB3* in these three transgenic lines compared to WT. **(C)** The relative DAB stain intensity in these three transgenic lines compared with WT calculated by IMAGE J software. **(D)** The relative NBT stain intensity in these three transgenic lines compared with WT calculated by IMAGE J software. **(E,F)** Accumulation of TuMV RNAs and CP in IL **(E)** and SL **(F)**. The relative intensity of the blot signal quantified by IMAGE J software is shown on the lanes. Bars represent the standard errors of the means from three biological repeats. A two-sample unequal variance directional Student’s *t*-test was used to test the significance of the differences (*P < 0.05; **P < 0.01; ***P < 0.001).

To further explore the biological function of *NbLHCB3* in TuMV-GFP infection, we obtained transgenic *N. benthamiana* overexpressing *NbLHCB3*, and detected the accumulation of TuMV in these plants. These transgenic plants contained the full-length ORF of *NbLHCB3* driven by the *35S* promoter of cauliflower mosaic virus (CaMV) and were obtained by agrobacterium-mediated transformation. Three independent transgenic lines (OE:*NbLHCB3* −4, −8 and −9) were identified with about three times increased expression of *NbLHCB3* compared to wild type *N. benthamiana* (Figure 6B) with no obvious developmental phenotype compared to WT (Figure 6A). In these plants, ROS levels were decreased (Figures 6A,C,D). After inoculation with TuMV-GFP, there were more fluorescent spots on the IL at 5 dpi than on the controls (Figure 6A) and fluorescence was also more extensive on their SL (Figure 6A). Blotting analysis showed a significantly increased accumulation of TuMV RNAs and CP on the three transgenic lines, further demonstrating that decreased ROS together with increased expression levels of *NbLHCB3* resulted in greater susceptibility to TuMV (Figures 6E,F).

## Discussion

Viral infections usually cause plant chlorosis, stunting, necrosis, or other symptoms (Shi et al., 2016). Chlorophyll pigmentation can be reduced following virus infection (Grisham et al., 2010), chloroplast structures and functions may be changed (Otulak et al., 2015; Rong et al., 2018), and the expression of nuclear-encoded chloroplast photosynthesis-related genes repressed (Mochizuki et al., 2014; Das et al., 2019; Zhao et al., 2019). All these suggest that viruses and chloroplasts are inextricably linked. Many viral proteins interact directly with chloroplast proteins thus linking chloroplasts to viruses (Ma et al., 2008; Sun et al., 2013; Balasubramaniam et al., 2014; Kong et al., 2014; Gnanasekaran et al., 2019). TuMV also associates with chloroplasts and its successful infection depends on the fusion of virus-induced vesicles with chloroplasts (Wei et al., 2013). In this study we found that chloroplast antenna protein NbLHCB3 was downregulated by TuMV infection and played roles in the interaction between TuMV and plants, providing further evidence of the association of chloroplasts with TuMV infection (Figures 1B,C).

Many studies have shown that viruses from different genera significantly downregulate chloroplast-related genes (ChRGs) including light harvesting genes (Bhattacharyya et al., 2015; Shi et al., 2016; Das et al., 2019; Zhao et al., 2019; Leonetti et al., 2021). The mechanism by which viruses regulate expression of these ChRGs is not well understood. One reported mechanism is RNA silencing, in which small RNAs derived from the viral genome or satellite RNAs target ChRGs for silencing (Shimura et al., 2011; Smith et al., 2011). We here found that the NIa protein of TuMV was responsible for *NbLHCB3* reduction, indicating a potential role for NIa in regulating chloroplast function (Figure 1D).

Light-harvesting chlorophyll *a*/*b* complex protein 3 interacts with PsbS and affects the macrostructure of photosystem II (Damkjaer et al., 2009; Kouřil et al., 2013; Pietrzykowska et al., 2014; Gerotto et al., 2015; Longoni et al., 2015). PsbS (NPQ4) plays a major role in activating the photoprotection mechanism known as “non-photochemical quenching (NPQ)” which dissipates chlorophyll excited states exceeding the capacity for photosynthetic electron transport (Fan et al., 2015; Gerotto et al., 2015; Correa-Galvis et al., 2016; Tibiletti et al., 2016; Sacharz et al., 2017). The latest research finds that PsbS can also activate photoprotection by some mechanism independent of NPQ (Redekop et al., 2020). OsPsbS-deficient rice generated more superoxide and hydrogen peroxide (Zulfugarov et al., 2014). Consistent with this, silencing of *NbLHCB3* here caused ROS accumulation and it seems possible that this was a consequence of the impaired NPQ in the *NbLHCB3*-silenced plants. Meanwhile, the *NbLHCB3*-silenced plants also had upregulated expression of genes in ROS production and downregulated expression of genes in the ROS scavenging pathway (Figure 3E). Although the mechanism by which *NbLHCB3* regulates the expression of these genes is not yet clear, there is clearly some indirect mechanism to cause ROS accumulation in the *NbLHCB3*-silenced plants.

Reactive oxygen species produced by RBOHs, and especially RBOHD, appear to be involved in plant immune responses (Sagi and Fluhr, 2001; Yoshioka et al., 2003; Torres et al., 2005; Asai et al., 2008; Lee et al., 2020; Ngou et al., 2021; Yuan et al., 2021). RBOHD is highly responsive to MAMP or pathogen treatments (Morales et al., 2016) and is negatively regulated through phosphorylation and degradation by ubiquitination (Lee et al., 2020). *NbRBOHC* is homologous to *AtRBOHD*. Here, the expression of *NbRBOHC* was upregulated in the *NbLHCB3*-silenced plants (Figure 3E). Co-silencing of *NbRBOHC* significantly impaired the inhibition of TuMV in the *NbLHCB3*-silenced plants, compared to other genes in the ROS production pathway, which indicates that *NbRBOHC* plays an essential role in the accumulation of ROS in the *NbLHCB3*-silenced plants (Figure 4).

It has been reported that light deficiency and photosystem impairment increased the susceptibility of *N. benthamiana* to TuMV infection, suggesting that both light and optimal chloroplast function influence virus infection by limiting the cellular resources needed by TuMV to establish replication complexes (Manfre et al., 2011). However, the effect of the photosystem on viral infection is likely to be complicated since many essential molecules, including ROS and various sugars that all play roles in plant defense, are produced from the photosystem (Bhattacharyya and Chakraborty, 2018; Littlejohn et al., 2021). Deficiencies of different components in the photosystem would have different effects on these molecules, which might result in plants showing different responses to pathogens. In our previous report, silencing of genes in the Calvin cycle limited rice stripe virus (RSV) infection (Bi et al., 2019). We supposed that the energy produced in the Calvin cycle was necessary for normal RSV infection. Here, silencing of *NbLHCB3* caused the accumulation of ROS, which was demonstrated to regulate TuMV infection negatively (Figures 3, 4). Both results suggest the different roles of photosystem products in response to viral infection and reflect the complicated functions of chloroplasts in the interaction between viruses and plants.

Reactive oxygen species themselves have also been reported to be harnessed by viral infection and transmission. Red clover necrotic mosaic virus and brome mosaic virus both harness host NbRBOHB-dependent O_2_^–^ for robust genome replication (Hyodo et al., 2017a,b). For TuMV, ROS is reported to be beneficial to aphid-borne spread (Berthelot et al., 2019; Guo et al., 2019). Taken together with the results of the present study, this suggests an interesting hypothesis that ROS accumulates in TuMV-infected *N. benthamiana* by down-regulation of *NbLHCB3* to inhibit TuMV infection, while the accumulated ROS enhances its transmission by aphids. We will examine this hypothesis next.

## Data Availability Statement

The original contributions presented in the study are included in the article/Supplementary Material, further inquiries can be directed to the corresponding author/s.

## Author Contributions

SQ, JC, and FY designed the experiments. SQ, XC, YZ, WC, XA, and SR performed the experiments and interpreted the data. SQ and FY drafted the manuscript. FY and JC revised the manuscript. All authors contributed to the article and approved the submitted version.

## Conflict of Interest

The authors declare that the research was conducted in the absence of any commercial or financial relationships that could be construed as a potential conflict of interest.

## Footnotes

1 https://solgenomics.net/

## Supplementary Material

Supplementary Figure 1 Sequence analysis and phylogenetic tree of LHCB homologs from different plant species. **(A)** Protein sequence alignment of NbLHCB3 and AtLHCB3. **(B)** Nucleotide sequence alignment of *NbLHCB3* and *AtLHCB3* cDNA fragments. **(C)** Phylogenetic tree of LHCB homologs from different plant species. NbLHCB3 is marked with red lines.

Supplementary Figure 2 Phylogenetic tree of *NbLHCBs* and relative expression levels of *NbLHCBs* in the TRV:LHCB3-inoculated plants. **(A)** Comparison of cDNA sequences and construction of phylogenetic tree of *NbLHCBs*. *NbLHCB3* is marked with red lines. **(B)** Relative expression levels of *NbLHCB* family members in the TRV:LHCB3-inoculated plants. Bars represent the standard errors of the means from three biological repeats. A two-sample unequal variance directional Student’s *t*-test was used to test the significance of the differences (^∗^*P* < 0.05).

Supplementary Figure 3 Northern blotting showing the accumulation of TuMV-GFP RNAs at 5 dpi in the plants where *NbLHCB3* was co-silenced with individual genes in the ROS production pathway, *AO*, *RBOHA*, *RBOHB*, *RBOHC*, and *RBOHE*. The relative intensity of the blot signal quantified by IMAGE J software is shown on the lanes.

Supplementary Figure 4 Northern blotting showing the accumulation of TuMV-GFP RNAs at 8 dpi in the plants where *NbLHCB3* was co-silenced with individual genes in the ROS scavenging pathway, *CAT*, *SOD(CuZn)*, *SOD(Fe)*, *SOD(Mn)*, *tAPX*, *GR1*&*GR2*, and *GR3*&*GR4*. The relative intensity of the blot signal quantified by IMAGE J software is shown on the lanes.

Supplementary Figure 5 The expression of *LHCB3* and genes in the ROS production and scavenging pathways were all downregulated in these co-silenced plants. **(A–E)** The expression of *LHCB3* and genes in the ROS production pathway (*AO*, *RBOHA*, *RBOHB*, *RBOHC*, and *RBOHE*) were downregulated in these co-silenced plants. **(F–L)** The expression of *LHCB3* and genes in the ROS scavenging pathway [*CAT*, *SOD(CuZn)*, *SOD(Fe)*, *SOD(Mn)*, *tAPX*, *GR1&GR2*, and *GR3&GR4*] were downregulated in these co-silenced plants.

Supplementary Table 1 Primers used in the experiments.

Supplementary Table 2 Accession numbers of *Nicotiana benthamiana* genes.
