# Supplementary figures and images for: Downregulation of Light-Harvesting Complex II Induces ROS-Mediated Defense Against Turnip Mosaic Virus Infection in Nicotiana benthamiana
Source: Front Microbiol. 2021 Jul 5;12:690988. doi: 10.3389/fmicb.2021.690988 (PMC8287655; doi:10.3389/fmicb.2021.690988)

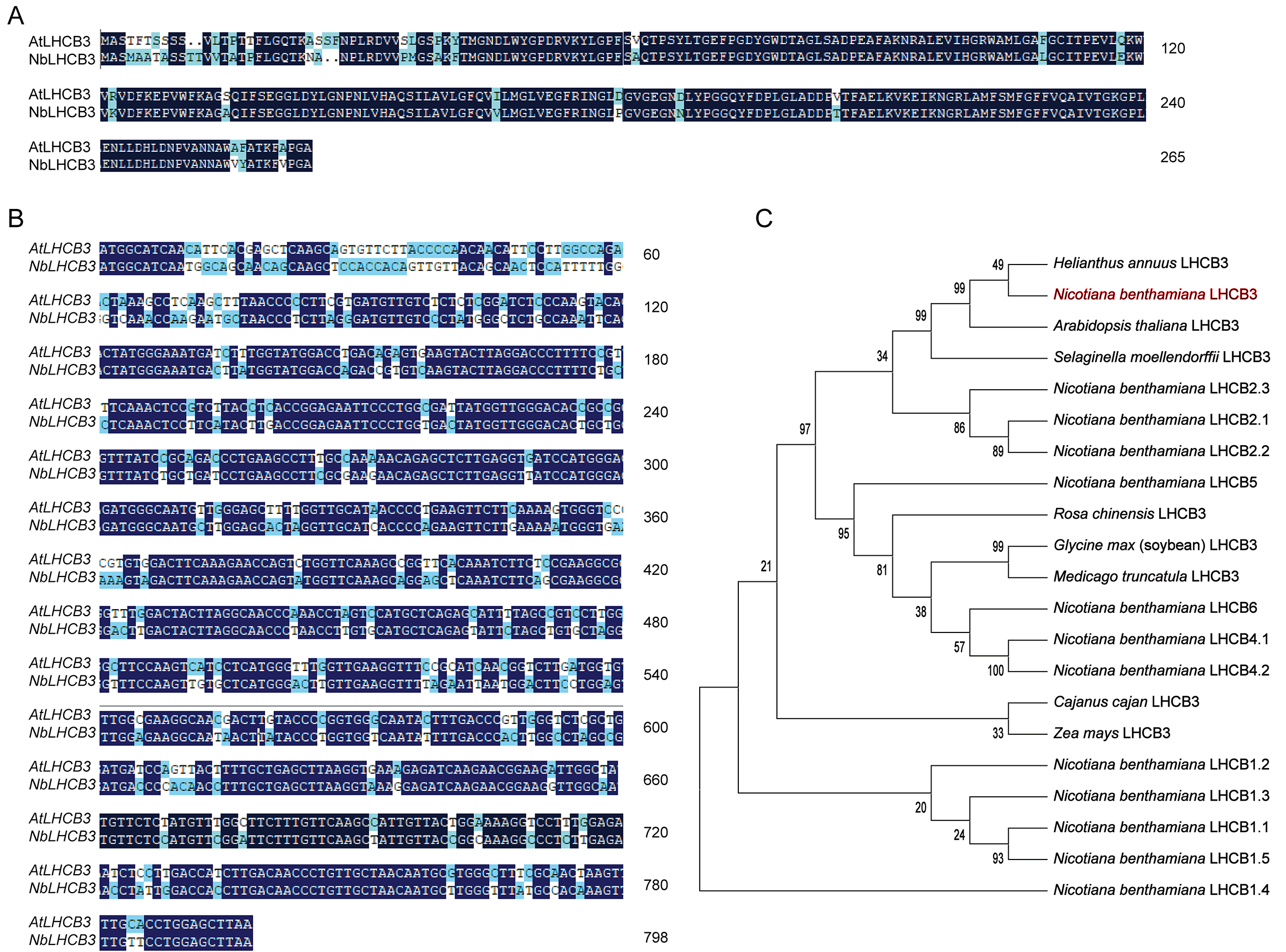

Supplement: Supplementary Figure 1 — Sequence analysis and phylogenetic tree of LHCB homologs from different plant species. (A) Protein sequence alignment of NbLHCB3 and AtLHCB3. (B) Nucleotide sequence alignment of NbLHCB3 and AtLHCB3 cDNA fragments. (C) Phylogenetic tree of LHCB homologs from different plant species. NbLHCB3 is marked with red lines. [file Image_1.TIF]

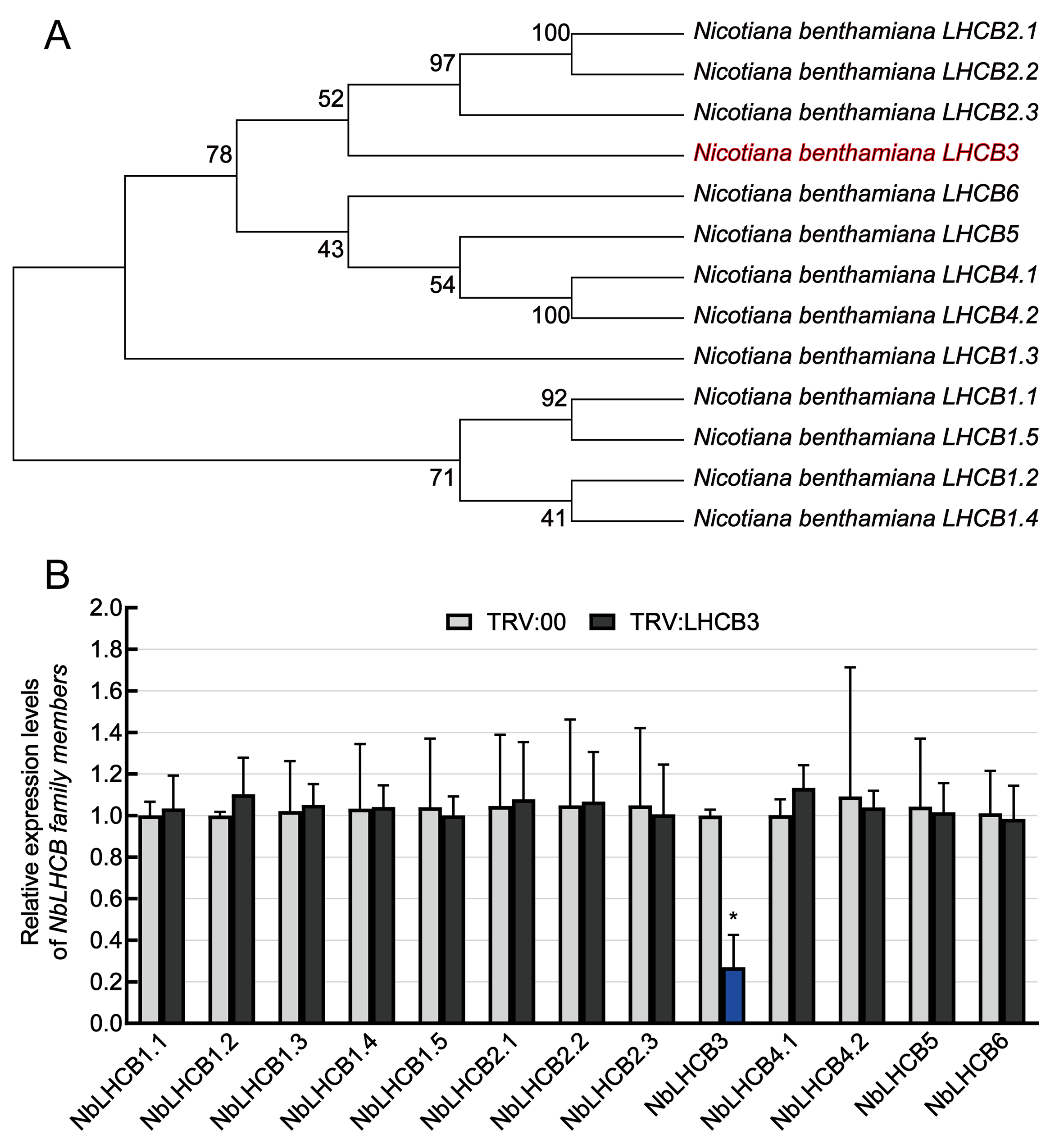

Supplement: Supplementary Figure 2 — Phylogenetic tree of NbLHCBs and relative expression levels of NbLHCBs in the TRV:LHCB3-inoculated plants. (A) Comparison of cDNA sequences and construction of phylogenetic tree of NbLHCBs. NbLHCB3 is marked with red lines. (B) Relative expression levels of NbLHCB family members in the TRV:LHCB3-inoculated plants. Bars represent the standard errors of the means from three biological repeats. A two-sample unequal variance directional Student’s t-test was used to test the significance of the differences (∗P < 0.05). [file Image_2.TIF]

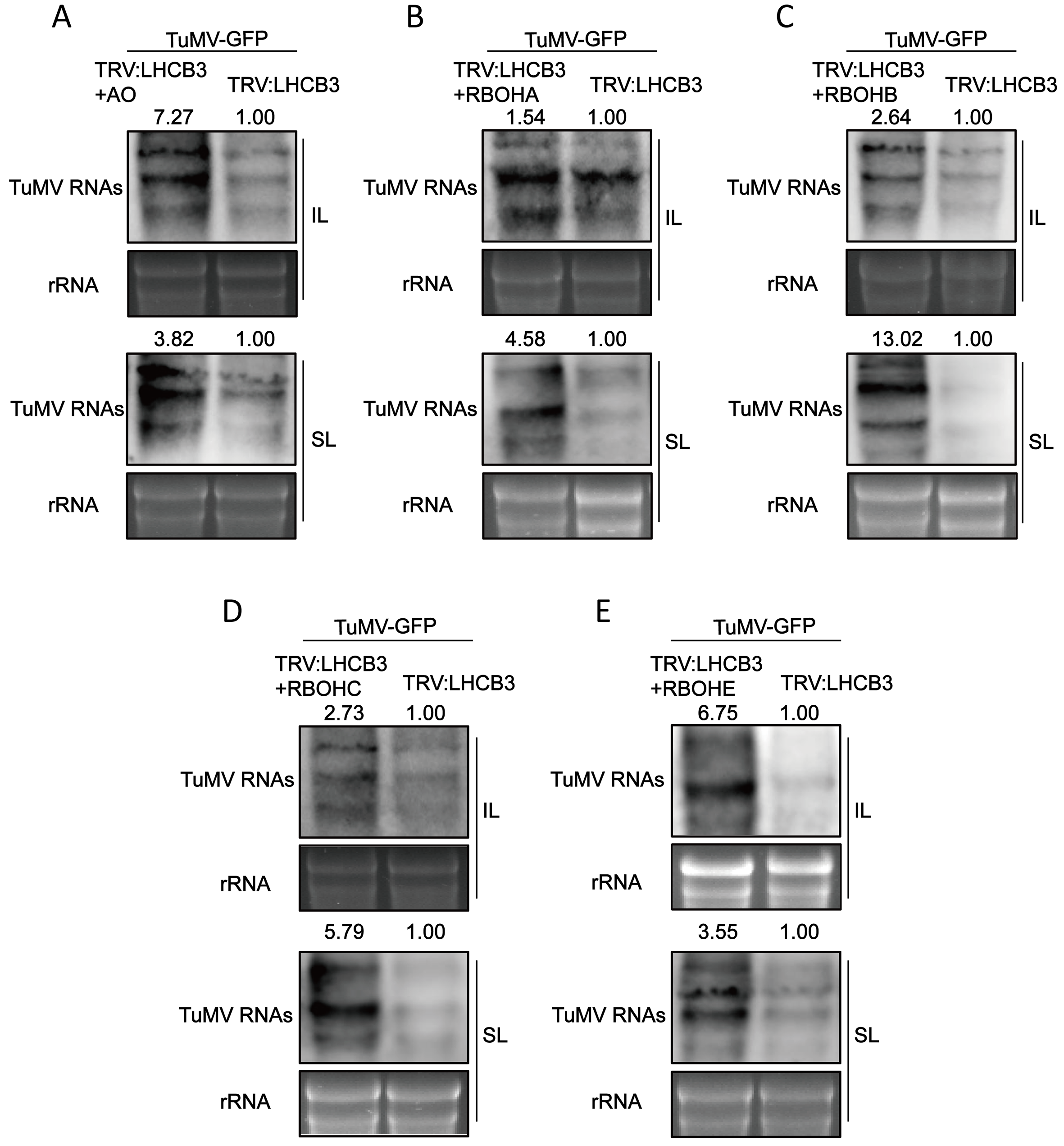

Supplement: Supplementary Figure 3 — Northern blotting showing the accumulation of TuMV-GFP RNAs at 5 dpi in the plants where NbLHCB3 was co-silenced with individual genes in the ROS production pathway, AO, RBOHA, RBOHB, RBOHC, and RBOHE. The relative intensity of the blot signal quantified by IMAGE J software is shown on the lanes. [file Image_3.TIF]

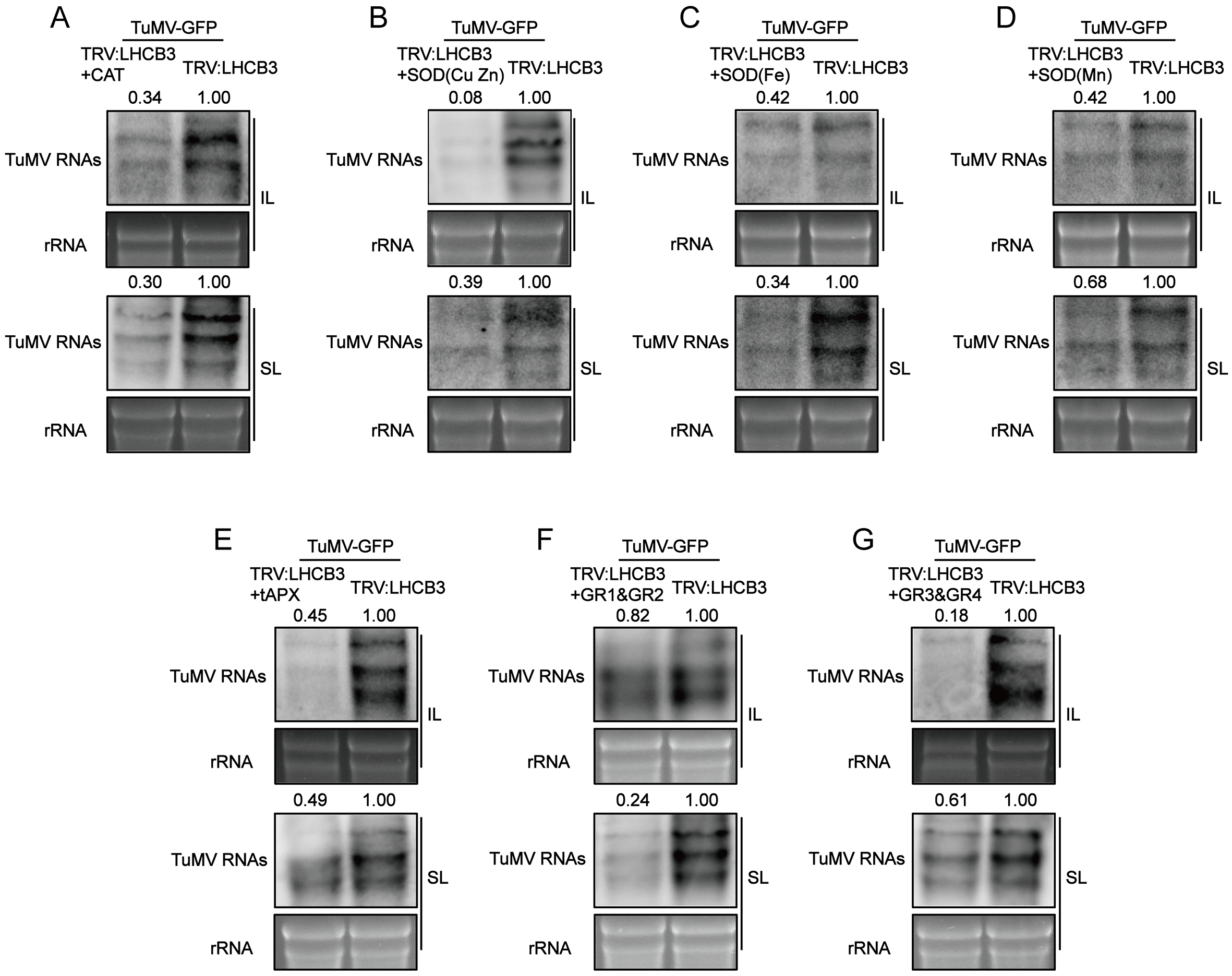

Supplement: Supplementary Figure 4 — Northern blotting showing the accumulation of TuMV-GFP RNAs at 8 dpi in the plants where NbLHCB3 was co-silenced with individual genes in the ROS scavenging pathway, CAT, SOD(CuZn), SOD(Fe), SOD(Mn), tAPX, GR1&GR2, and GR3&GR4. The relative intensity of the blot signal quantified by IMAGE J software is shown on the lanes. [file Image_4.TIF]

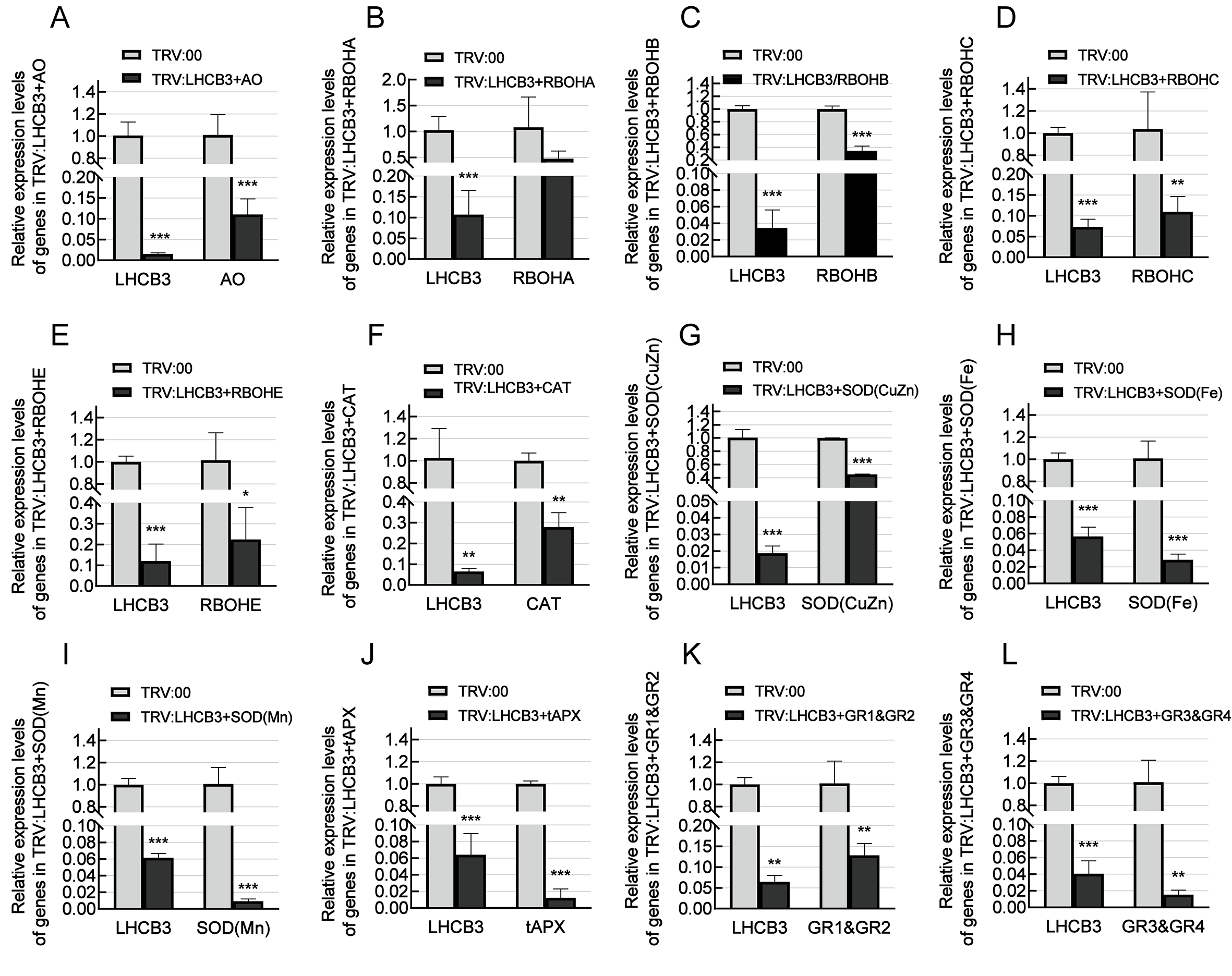

Supplement: Supplementary Figure 5 — The expression of LHCB3 and genes in the ROS production and scavenging pathways were all downregulated in these co-silenced plants. (A–E) The expression of LHCB3 and genes in the ROS production pathway (AO, RBOHA, RBOHB, RBOHC, and RBOHE) were downregulated in these co-silenced plants. (F–L) The expression of LHCB3 and genes in the ROS scavenging pathway [CAT, SOD(CuZn), SOD(Fe), SOD(Mn), tAPX, GR1&GR2, and GR3&GR4] were downregulated in these co-silenced plants. [file Image_5.TIF]
